# Systematic review on ensuring the global food security and covid-19 pandemic resilient food systems: towards accomplishing sustainable development goals targets

**DOI:** 10.1007/s43621-022-00096-5

**Published:** 2022-08-31

**Authors:** Keerththana Kumareswaran, Guttila Yugantha Jayasinghe

**Affiliations:** Department of Agric. Engineering, Faculty of Agriculture, University of Ruhuna, Matara, Sri Lanka

**Keywords:** Build back better systems, Covid-19 pandemic, Food system resilience, Global food security, Sustainable development goals, System thinking

## Abstract

Covid-19, one of the most critical and widespread global pandemics, has resulted in extraordinary risk corollaries engulfing millions of people's lives and has caused an unprecedented economic downturn while amplifying food insecurity. A systematic review of 132 scientific communications was performed over a 15-year period, using articles from the ScienceDirect and Web of Science databases (2006–2021). In addition, 24 policy briefs, country papers, and publications from the UN, WHO, FAO, and OECD were cited. The aim of this paper is to provide a comprehensive review of existing literature on the adverse effects of the Covid-19 pandemic on agricultural food systems, as well as potential strategies for building robust, resilient, and sustainable food systems to ensure global food security, safety, and endeavors regarding future global emergencies, as well as new research policies while achieving SDG targets. This would fill a research gap while also having long-term implications for health, agricultural, and food resilience policy development in a rapidly changing world. Covid-19 demonstrates how human, animal, and environmental health are all interconnected, emphasizing the need for one health legislation and a paradigm shift in planetary health. Furthermore, it identifies potential mechanisms for rebuilding better systems by shifting priorities toward policy coherence, innovative food system governance, re-engineering market access, and nexus thinking in the food system approach. According to our findings, the COVID-19 posed unavoidable impediments to achieving SDG targets for food security and household poverty.

**Graphical abstract:**

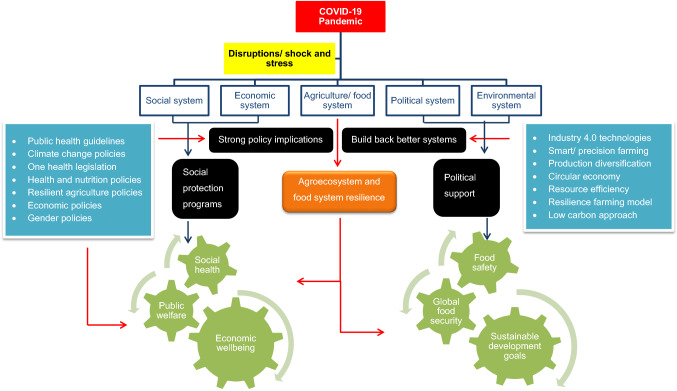

## Highlights

- COVID-19 pose acute shock and persistent threat on agri-food supply chain systems disproportionately affecting developing nations.
- World in off track in accomplishing SDG goals in alleviating poverty, hunger, and malnutrition.
- Require diversified robust food production, distribution, and supply chain networks based on agroecology to combat pandemic crisis.
- Shifting to system thinking, holistic food system approach, and build back better system results in food system resilience.
- One health legislation, eco health/ planetary health paradigm to be at the centre of new global emergencies.

## Introduction

### Covid-19 and history of global pandemics

Approximately 60% of diseases that emerged between 1940 and 2004 are infectious [1]. Three out of every four new diseases are caused by animals [2]. The close proximity of domestic, wild, and human animals resulted in the spread of zoonotic diseases [3–8]. It is predicted that unless agricultural/farming intensification and large-scale unsustainable animal outputs are prevented, another strain of virus will emerge in the future, with mutations resulting in different strains [1, 9]. The extensive and indiscriminate use of chemical fertilizers, growth regulators, and antibiotics would result in drug-resistant pathogenic viruses and super bacteria [10].

Prior to the arrival of Covid-19, there had been relatively few notable epidemics and disease outbreaks throughout human history [11]. Below is a list of a few of these dreadful contagious diseases, along with information on their causes of outbreaks and probable consequences (Table 1). Corona infections were first found in China in December 2019; an outbreak in other countries is projected to begin in February 2020 [29]. The World Health Organization quickly declared it a pandemic that required a public health emergency on March 11, 2020 [20, 43]. The likelihood of widespread distribution has increased as a result of global transit, socioeconomic, and political interactions [16, 24, 44].

**Table 1:** Global pandemics, their causes of breakout, and probable consequences

Global pandemics	Year of outbreak	Root causes of outbreak	Potential impacts	References
Bubonic plague	Fourteenth century	Spread in Central Asia, Europe, and China	Brought radical changes in urban infrastructures Social, cultural, and political renaissances because of massive economic and social upheaval One third of Europe's population was killed in the east, and feudalism came to an end 25 million infected in China	[12, 13]
Spanish flu/influenza pandemic	1918–1920	Disastrous virus pandemic	Killed more than 50 million populations One third of the global population then was infected Questioned human health, dragged down global economies from progress for long term Messed up food supply chains	[14–17]
Severe Acute Respiratory Syndrome (SARS-CoV-1)	2002–2004	Due to rapid infrastructure development in Asia along with higher rates of demographic and geographic transitions Outbreak in China and spread to 20 countries	Affected food security Disrupted the lives of vulnerable population including poor households, women, children, smokers, and elderly people 10% lethality rate	[1, 12, 14, 18–21]
Avian influenza (H5N1)/ African swine fever	1997 in Hong Kong 2004–2006	Highly virulent pandemics originated from pigs and chicken Large scale unsustainable animal productions Relatively low lethality but strong infectivity	Fatality rate 60% Had direct strong impact on food production and greatly reduced animal-based product output Sharp decline in agri-food production and food insecurity emerged	[10, 22–25]
Middle East respiratory syndrome MERS-Cov	2012	Detected in Saudi Arabia Spread to 27 countries Short incubation period High lethal rates	Affected food security particularly disrupting the lives of vulnerable population HERA protocol stemmed out Lethality rate 35% Devastated agriculture production and allied labor sector	[1, 7, 12, 18–21]
Swine flu/ influenza (Influenza A H1N1)	2009 outbreak in Mexico	Human-to-human transmission like seasonal flu Respiratory disease caused by viruses that infect the respiratory tract of pigs, resulting in nasal secretions, cough, and decreased appetite	Had killed over 50 lakhs people globally 18,449 deaths in 2010 Most serious complication of the flu was pneumonia People with chronic medical conditions, children less than 5 years, persons above 65, and pregnant women were at increased risk	[23, 26–28]
Ebola hemorrhagic fever	1976 in West Africa 2014–2016 West Africa	Unsustainable agriculture practices Increased interaction between infected animals and human due to deforestation	Mortality rates up to 43% in West Africa Affected food security particularly disrupting the lives of vulnerable population including poor households, women, children, and elderly people Ebola in Europe and North Africa resulted in child labor, child abuse, gender violation, domestic violence, and teen pregnancies Increased the price for rice in countries like Guinea, Liberia, and Sierra Leone by 30% and cassava by 150% in Liberia while lowering fertilizer use in West Africa In Africa killed about 10 000 people	[7, 12, 13, 19, 21, 25, 29, 30]
Marburg virus (MARV)	Since 1967	Unsustainable agriculture practices Egyptian fruit bats are hosts *(Rousettus aegyptiacus)*	Responsible for several outbreaks of highly fatal hemorrhagic fever to human and primates 90% fatality rate Highly contagious and manifests symptoms of high fever, diarrhea, vomiting, and severe bleeding in the bod	[30–32]
Yellow fever	Since seventeenth century 1948 1960–1962 1985–1995 2016–2018	South America and Sub-Saharan Africa Mosquito-borne disease Outbreak when African slaves imported as human cargo	200 000 active cases and 30 000 deaths annually 90% of cases reported in Africa	[25, 33–35]
Smallpox	Since third century in Europe 1977 last naturally occurred case reported in Somalia	Caused by two variants Variola major and Variola minor Hemorrhagic smallpox was severe	Cause potentially lethal interstitial pneumonitis as well as tubule interstitial nephritis Cytopathic effects cause death Extensive scarring of skin and left blind Fatality rates exceeded 30% Children below 10 years were more susceptive 500 million people killed in last century	[36–38]
Lyme disease		Transmitted to humans through the bite of a tick infected with various B. burgdorferi sensu lato bacteria Outburst in North America due to decline of red fox Unplanned suburban development Forest clearing	240,000 to 440,000 new cases diagnosed every year in US Post treatment Lyme disease syndrome for 10–20% individuals: fatigue, musculoskeletal pain, and neurocognitive complaints Erythema migrans/early-stage acute skin infection Manifestation of late Lyme disease such as arthritis, encephalomyelitis, or peripheral neuropathy Higher health care cost	[1, 23, 39–42]

The Covid-19 pandemic is a consequence of climate vulnerability with long-standing historical roots in antagonistic anthropogenic activities [45]. SARS-Cov-2 and bat coronavirus have genomes that are 96.2% similar, according to scientific research [18, 34]. There is no proof to support the outbreak, dispersal, or transmission of SARS-Cov-2 through food, despite the fact that MERS and SARS corona virus outbreaks were shown not to be spread through food [34].

### COVID-19: contemporary issues in global sustainable development

The recombinant SARS-CoV-2 Corona virus, also known as Covid-19 [43, 46], has put a significant burden on the world's health care system and sparked worries about the resiliency of agri-food systems [11, 47, 48]. Due to food scarcity, insecurity, and malnutrition, it posed a serious threat to agriculture and the global food supply chain systems [49]. The effects of increased poverty and hunger are clearly visible in many nations around the world, having a devastating impact on low-income households, particularly those that are headed by women, low-wage workers, and those who reside in urban settings [4, 50–52].

The Covid-19 pandemic was widespread and unpredictable, and as of November 7th, 2021, 5,027,183 deaths had been reported [7, 53, 54]. Unprecedented economic downturn [7, 55] and disruptions in all enterprises and supply chains [4, 17, 43] have been brought on by it.

In terms of the pandemic’s effect on GDP globally, several projections have been made. IMF, World Bank, and OECD forecasts assert that a recession will hit the world economy in 2020, resulting in 495 million people being forced to work without pay and a GDP loss of 3 to 7.5%, with 2021 GDP growth forecast to range from 2.8 to 5.8% [56]. Major international economies like China, the United States, and Europe will be affected by the World Bank's projection of a 32% decline in global trade [22].

Covid-19 adversely affected developing countries’ food supply chains disproportionately because their economies are heavily dependent on agricultural imports and agribusiness [21, 57, 58]. Because of the unprecedented Covid-19 situation, it is clear that improving the ability of agriculture and food supply systems to withstand external disturbances is essential. To satisfy consumer demand, a regionally self-sufficient economy that is socially inclusive and interconnects conventional and commercial agriculture systems is necessary [59]. A strategy for inclusive agriculture similar to that used in Poland [60] was also recommended by Ambros and Granvik’s [60].

Diverse food production systems were gradually replaced by intense industrial monocultures, and consumer food behavior and practices caused diet patterns to change from a traditional diverse diet that was safe and healthy to highly processed, energy-dense foods that were deficient in micronutrients [61]. Mora et al. [62] identified the potential to reduce the expansion of agricultural land by changing the global diet to healthier patterns [62]. However, the public’s perceptions had drastically changed since the advent of Covid-19. The priorities of people have evolved as a result of life lessons learned, and they now consume differently and shop differently [63]. By repairing damaged ecosystems and sustainably managing air, water, and land, Covid-19 has given humanity the chance to atone for its transgressions [13, 50, 64]. In order to rebuild better systems, post-pandemic governance had come to realize the necessity of interaction and inter-functioning among agriculture, public health, environment, policy framework, politics, economy, and research and development [12, 65].

## Methodology

### Systematic literature review

A systematic literature review was carried out to develop a comprehensive conceptual framework for ensuring global food security and developing innovative pandemic resilient food systems in order to accomplish SDG targets.

### Identification

Original research papers and review articles were obtained from the ScienceDirect and Web of Science databases, which are well-known for their high-quality and higher peer-reviewed paper indexes. The title search was conducted as follows, using keywords and the Boolean operators [66].

“Covid-19 pandemic” AND (“global food security” OR “food system resilience” OR “global pandemics” OR “agroecosystems” OR “build back better” OR “systematic thinking” OR “potential response mechanisms” OR “policy instruments”).

The initial search yielded 7462 and 191 items for ScienceDirect and Web of Science, respectively. In addition, 24 policy briefs, action plans, and publications from the UN, WHO, OECD, FAO, and the Central Bank of Sri Lanka were referenced.

### Screening

Once the search period was narrowed to 2006–2021 and only peer-reviewed English papers were evaluated, the total number of articles decreased to 4926 for ScienceDirect and 168 for web of science, respectively.

### Eligibility and inclusion

**Fig. 1:**
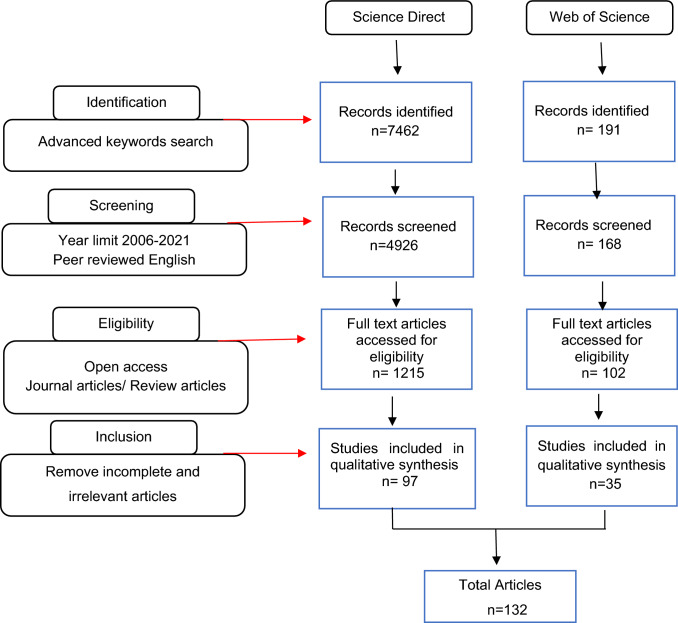
Systematic article selection procedure

The publications were chosen for further examination if they are open access research and review articles. There were 1317 full text articles, and all selected entries were imported to JabRef software and then exported to excel file as CSV data after removing duplicates. Further the studies that were not relevant to Covid-19 pandemic but were relevant to global food security or vice versa were excluded. After screening the titles and abstracts, the complete text of 132 papers were examined, and all 132 articles satisfied all inclusion requirements. Figure 1 outlines the flow diagram of the systematic article selection procedure used in the study.

## Potential impacts of Covid-19 on global food production systems

### Food security and nutrition

**Fig. 2:**
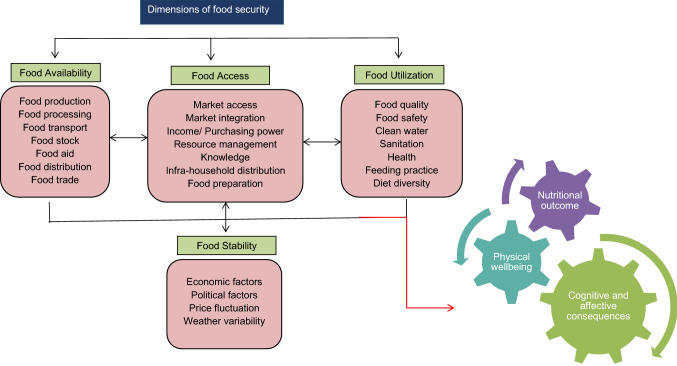
Dynamics of food security and nutrition. Source: [16, 65]

Food security is defined as the economic and physical accessibility of all people to sufficient amounts of wholesome food to meet their dietary needs and maintain an active and healthy lifestyle [67–69]. According to the benchmarking pillars for the global food security index [29], food security has six dimensions: availability, access, utilization, stability, quality, and safety, as shown in Fig. 2 [16, 65].

Due to malnutrition and a lack of micronutrients, Covid-19 and its effects have had a number of detrimental effects on food safety and nutrition [22]. People with diabetes, hypertension, and obesity are more susceptible to COVID-19 [5, 70]. The majority of the time, this condition is seen in developed or developing nations with middle- to high-income levels, which are suitable for urban settings. The risk of diet-related communicable and non-communicable disorders is eliminated by a safe diet.

There are two possible scenarios related to food and nutrition intake.

1. Inadequate nutritional intake: Inadequate macro and micronutrient intake (protein, vitamins, and minerals), which impedes children's growth and development, resulting in stunting and wasting [71].
2. Poor food consumption: high calorie nutrient food, cheap and low-quality fast food, reduced consumption of perishables and pulses, highly processed food, high sugar food, higher consumption of sodium, saturated and trans-fats, and high energy fatty acids all contribute to obesity, diabetes and are risk factors for Covid-19 mortality [5, 48, 68, 72, 73].

### COVID-19 impacts on food system dynamics and dimensions of food insecurity

**Fig. 3:**
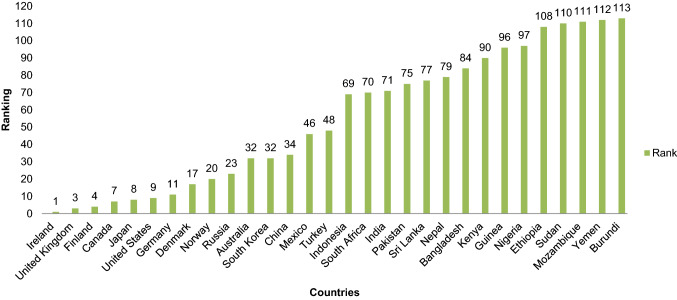
Global Food Security Index Ranking 2021. Data source [74]

According to Global Food Security Index developed based on 59 indicators considering the issues of food affordability, availability, quality and safety, and natural resources and resilience across 113 countries; global food security is deteriorating over decade primarily due to climate variability and intensive farming further, the conditions were aggravated by Covid-19. The graph (Fig. 3) represents the global food security index ranking of the year 2021, where Ireland secured top position with highest level of food security. While Sub-Saharan African countries are the most food insecure; spotting Mozambique, Yemen, and Burundi at the bottom [74]. Ireland was successful in maintaining higher nutritional standards with adaptation policies at lower food cost while tackling the food affordability and quality. It had made an extensive investment in agricultural R and D and instilled food safety nets curbing food inequality and food loss.

The Covid-19 epidemic has exacerbated global food insecurity [75] and has significant consequences on agri-food system channels [21, 76] particularly in developing countries than in developed countries [70, 77]. Food system inequalities increase food insecurity cases from 83 to 132 million globally, while in the United States, it has tripled since 2019 [78]. The World Food Program predicted that 265 million people will be suffering of severe food insecurity by the end of 2020, up from 135 million before the Covid health crisis [17]. The results of the scenario analysis explicitly demonstrate the prevalence of chronic food insecurity and the highest level of susceptibility to food supply shocks in Africa, with pandemic consequences cascading [58].

A study that covered 136 nations found that the price of common foods like bananas, eggs, rice, and bread increased as a result of corona by anywhere from 2% in Bangladesh to 23.5% in Nigeria [49]. The combined effects of Covid-19 and government travel restrictions increased demand for some mainstream goods while decreasing demand for others, such as luxury goods. Price changes have resulted from citizens and some governments stockpiling necessities as a result of the lockdown and growing anxiety about future shortages [79]. This panic consumption pattern increased unwarranted price volatility and stressed the supply, distribution, and production chains for food [59]. Furthermore, current financial instability has reduced income, decreased purchasing power, and altered consumption patterns [49, 80]. Panic purchasing, stockpiling/ hoarding behavior and a dramatic increase in food demand caused short-term issues like temporary food scarcity, restricting food supply, particularly for long-lasting foodstuffs [59, 81]. But decreased income caused long-term problems [19, 21]. Border closures resulted in a manpower shortage, notably for migrant workers, as well as a lack of farm inputs, putting labor-intensive products like perishable fruits and vegetables at jeopardy [29, 82]. The absence of monitoring, commissioning, and maintenance services for machines, production and distribution delays, problems with quality assurance, underproduction of necessities and overproduction of luxuries, closures of restaurants, five-star hotels, and bakeries, and decreased market prices are examples of indirect effects [83, 84]. Price fluctuations brought on by the economic downturn significantly lowered the costs of high-value commodities; in particular, the cost of international meat and dairy products tumbled by 7–18% and 4–7%, respectively [56].

A potential strategy to maintain food security is to include underutilized crops that are frequently grown on marginal lands in agricultural production [85], as well as nutri-cereals and smart foods that are adaptable to a variety of environmental contexts, have higher nutritional quality, functional foods with bioactive ingredients/antioxidants, are resilient to environmental stress, pests, and diseases, have enhanced water and nitrogen usage efficiency, and have improved agroecological traits [18, 85].

Covid-19 policy response portal had tracked five major trends and implications in food system security based on the data collected across 18 countries in Asia, Europe, and Africa; such as restrictions on urban food trade (e.g. wet markets closed in Burkina Faso), wide adoption of innovative contactless payments, government mechanisms on protecting consumer livelihoods, comparatively less support to agriculture, and exclusion of agriculture ministers from response plans in national level [86].

### Environmental risks, socio-economic impacts, and vulnerabilities

In UN 2020, the governing body of the European Union in Brussels declared that “industrial agriculture increases the risk of future pandemics and must be addressed” [61]. Recent IPCC assessment reports claim that the current industrial food production systems are unsustainable and put the world's ability to feed 10 billion people by 2050 in jeopardy [1, 87–89].

The transformation to agroecology is a difficult and risky process. Diplomatic negotiations to increase agroecosystem resilience require strategic planning, reliable investment financing, methodical analysis of long-term green investments, and thorough policy options evaluations [90, 91]. Table 2 shows potential methods for increasing agroecosystem resilience to meet various dietary needs and food demands.

**Table 2:** Potential practices to enhance agro-ecosystem resilience to cater the diverse food demand and dietary requirement

Strategies	References
Sustainable agriculture (environmental and nutrient conscious)	[65, 67, 78, 85, 92, 93]
Conserve and breed agrobiodiversity	[9, 78, 85, 94–98]
Development of local/ regional crop and livestock production system	[47, 61, 88, 95, 99]
Develop agriculture-based cottage industries and entrepreneurship	[88]
Empowering local community especially women, agriculture labor, and vulnerable population	[13, 99]
Promote natural mechanisms to regulate biogeochemical cycles and pest management	[97, 100]
Biological pest management	[101, 102]
Low carbon agriculture/economy	[13, 103–106]
Crop and land specific agronomic practices	[107]
Improved plant genetics and crop varieties	[87, 92, 108, 109]
Renewable energy use (biofuel, solar, wind)	[13, 108]
Innovative soil management and enhanced soil biodiversity	[50, 64, 65, 99, 107, 110, 111]
Good governance of natural and human systems	[77]
Decentralized farm structures, improved working condition, anddecent wages	[83]
Incorporate traditional knowledge to modern technology through promoting cultural diversity	[13]
Avoid products/ ingredients of higher environmental footprint, over processed	[99]
Improved cold chain storage and transport facilities	[112]
Improved seed/grain storage to boost productivity	[78]
Enhance product value addition	[113]
National policies to prohibit land degradation and soil restoration	[67]
Increased share to local market and alternative distribution channels	[113]
Native plant (drought tolerant, nitrogen fixing) species and local farming practices	[8, 98]
Multi-stakeholder involvement	[60]
Generate, update, and maintain data on on-farm agrobiodiversity to measure performance and resilience of the farm and identify room for development	[96]
Socio-technological innovations, innovation and resilience policies, and investment on R&D	[96]
Transferring the intrinsic value of farming through incorporating youngsters	[60]
Develop productive, multifunctional, and diversified organic farming	[60, 72, 105]
Management practices to enhance mineral availability	[107]
Development of climate change, hydrological as well as crop models that assist in decision making and predicting pandemics	[100]

**Fig. 4:**
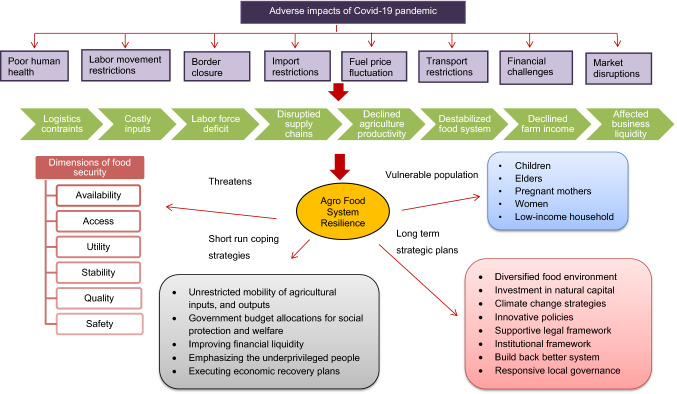
Impacts of Covid-19 food system dynamics on six dimensions of food security, and coping strategies to tackle the pandemic. Source: [6, 8, 22, 51, 82, 84, 114]

Figure 4 depicts the impacts of Covid-19 on agri-food system resilience specifically on agricultural productivity, supply chain, and global food security while revealing the potential measures to deal with the pandemic in short and long run. Among the adverse impacts, labor shortages mostly impacted agricultural operations, notably seasonal production, distribution of agricultural raw materials and finished goods, and livestock in labor-intensive agricultural production systems such as high-value crops, fisheries, and meat products [21, 58, 115]. Due to prohibited mobility, there is surplus labor in one area of the state and an insufficient labor pool in another, resulting in variable labor charges [53].

Based on the negative experiences of Covid, the EU recognized the need for business transition, social ethics, multiple local business trade chains, reduced reliance on international markets, quality standards, establishing and strengthening agriculture policy frameworks, and increased resilience in food production systems [75].

Lin and Zhang’s [14] study findings provided insights on the effects of the pandemic on agricultural exports in China, confirming that although international exports were sharply declined (by 17.2% in Feb 2020 compared to 2019) during the pandemic, exports of certain essential products such as grains, herbal items, and oil increased more than the prevailing levels with increased potential demand in the global market [14]. However, exports of horticulture, edible fungus, and livestock goods have declined significantly [21]. Agricultural trade in Canada is predicted to decline by 12–20%. Exports of livestock, pulses, and horticulture are falling off most noticeably, and revenues are falling as a result of the weaker purchasing power of the importing nations [55].

Trading strategies (Table 3) have been put into place by international countries [29]. The prices of staple foods like rice, wheat, and oil will significantly increase as a result of these moves by the world's major exporting countries [55]. The Just in Time supply model can improve the resilience of the food supply chain under normal conditions because there will always be an output with relatively low stocks, but it cannot be used in pandemic situations because of exogenous supply and demand shocks. Short-term interruptions were caused by increased food demand [84]. Agricultural policies that prioritize industrial export agriculture systems have significantly harmed subsistence food production and regional markets. Due to international border closures and trade prohibitions, **this proscribed food security** [52]. Low-middle income countries depending on the subsistence farming were interrupted due to pandemic than modern farms as evidenced in Ethiopia, disrupted supply of inputs, and vegetable supply with poorly developed infrastructures [22].

**Table 3:** Trading strategies implemented by world countries to limit pandemic outbreaks and guarantee local food security Source: [55, 81]

Countries	Measures
India	Suspension of new trade contracts for rice by traders, cancelling of license and permits for export
Cambodia	Prohibited the export of rice and fish
Thailand	Prohibition of chicken eggs export
Singapore	Elimination and reduction of tariffs and duties for essential goods/ agriculture products
Indonesia	Suspended import certification for onion and garlic
Turkey	Permit control/export control for lemon
Vietnam	Rice export control/stopped issuing new export certificates Control over the exports of flour, sugar, potatoes, and sunflower oil
Russia	Set limit on grain export to conserve food reserves
Kazakhstan, Serbia,	Control over the exports of flour, sugar, potatoes, and sunflower oil
European Union	Allowed imports of fruits and vegetables from India after assuring food safety and plant health standards via online certificates

During Covid, livestock farms are challenged with breeding, slaughtering restrictions, acquiring feed stocks, fodders, medicine, labor, machinery and associated high costs as well as marketing of final animal products like eggs, milk, and meat [115]. About 38.5% of Chinese farms were affected by significant logistics issues as a result of lockdown measures that resulted in price hikes [13, 32, 116].

Research on the Covid-19 aftermath has shown that reviving the agriculture sector in Nepal has been 3–5 times more effective in eradicating poverty than expanding other sectors [48, 114]. This is because it can increase production, create large numbers of jobs, reduce food insecurity, and is crucial for increasing agricultural production and reestablishing global food supply systems.

With the pandemic outbreak and the ensuing export–import controls, there was a positive transformation because the import of inorganic fertilizers was significantly decreased. It led to a rise in the use of organic fertilizers, which significantly reduce the harmful effects on the environment through resource efficiency, waste recycling, nutrient recovery, and circular economy. Hence, improves edible crop quality while preserving soil health as a result [50, 64].

Amuda [117] contends that in order to reduce the socioeconomic effects of COVID-19 on the Nigerian economy and to diversify the economic return through agriculture, a shift away from oil revenue to small-scale cocoa plantations (maize, cassava, millet) and yam must be made. Rediscovered interest in short supply chains, especially for local breeds and their products, is another positive outcome of Covid-19. Italy is seeing a rise in interest in the Alpine ark, Reggiana cattle and cheese, as well as nature-based travel [9]. The Netherlands is also witnessing a similar trend.

### Social protection programme to address global food insecurity

**Fig. 5:**
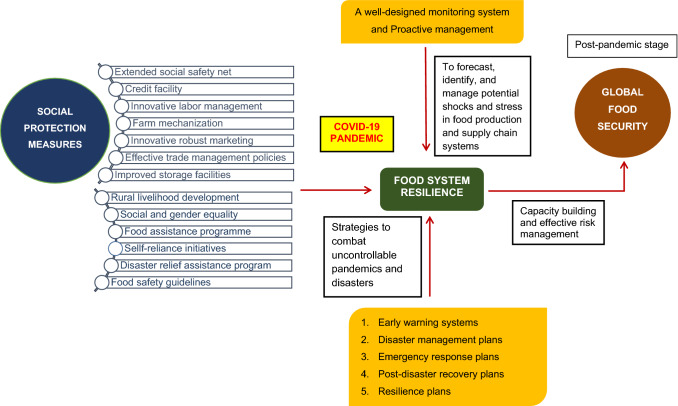
Potential mechanisms in addressing Covid-19 pandemic in accomplishing food system resilience. Sources: [17, 50, 63, 77, 119]

Social safety net programs, whether public or private, are known as the mechanism designed to support families' or individuals’ minimum consumption standards to meet daily nutritional requirements and are absolutely necessary in times of economic instability or natural disaster [118]. More than 195 nations around the world implemented social protection measures during the Covid-19 pandemic to shield citizens from decreased household income [22]. To maintain the efficiency of the food supply and production networks, several food aid programs are being established [77]. Figure 5 describes the potential mechanisms to protect social wellbeing and public welfare in addressing Covid-19 pandemic in accomplishing food system resilience. However, attempts to address covid problems were not met with satisfying results [63]. Table 4 shows the different potential response mechanisms in practice across the nations to combat pandemic.

**Table 4:** Different potential response mechanisms employed across the world countries to tackle Covid-19 pandemic

Potential Response Mechanisms	Country	Purpose	References
Grace periods for loan repayment, dampen interest rates, additional lending capacity to Farm Credit Canada	Canada	To reduce the financial burden of framers to ensure production and support them to face challenges in Covid uncertainty context	[59, 63, 82]
Corona virus food assistance program	USA	To ensure food access in times of pandemic	[63]
Shopping basket program, Incentive/ insurance scheme for vegetables	China	Ensure farmer’s income and protect them, stabilize agriculture production and supply	[120]
Multi-year joint resilient program	Somalia		[78]
Safety net program, purchase for progress program, food banks, emergency food pantries	Ethiopia	Chronically address insecurities	[78]
Nutrition sensitive cash transfer program		Enable equal access to health and nutritious food; especially considering vulnerable population	[22, 77]
One million kitchen garden plan	Kenya	To ensure healthy diet combat the food crisis caused by the pandemic Promote women empowerment	[57]
Digital agriculture/e-commerce platform: e-Soko, FarmCrowdy	Ghana, Nigeria China	Provide online farm management and technical and extension services	[49, 57]
Slow food gardens	Africa, Uganda, Malawi, Kenya Tanzania	To make communities resilient with diverse food (home, school, and community gardens)	[52]
Financial support, provision of subsidies, technology guidance,field management, and effective supply of critical inputs Cereal bag program Food basket program	China	To stabilize agriculture production and supply and compensate farmers for income loss	[19, 115]
Publications on Covid-19 and food safety guidelines for food companies, best practices for food shops, restaurants, delivery services, food safety, and hygiene advisory	Brazil, USA, China UK, Portugal, India	To incorporate food health, hygiene, and safety measures, as well as personal hygiene, to prevent future dangers	[34]
One planet sustainable food system programme	UN member states (Argentina, Switzerland, Tunisia)	To transform to responsible production and sustainable consumption and promotes holistic and inclusive policy making food systems approach	[121]
C40 cities food system network	Denmark, Barcelona Tokyo	To curb food related emissions through sustainable consumption, reduce food loss, support healthy plant-based diet in cities	[121]

## Building innovative, resilient, and sustainable food system in a transforming world to face future pandemics

Building food resilience in an environment that is changing is a dynamic and challenging task. The ability of a food system and its components “at multiple levels to provide sufficient, appropriate, and accessible food to all in the face of various and even unforeseen disturbances” is how it is defined [49]. It retains the same functions, structure, identity, complex relationships, and feedbacks while absorbing the shocks and disturbances [69, 99]. In order to create sustainable food systems, a resilient food system has five capacities for managing stress, risk, and shock. It speaks to the ability to anticipate, stop, absorb, adapt, and transform current food system risk [78].

Covid-19’s rapid emergence has both direct and indirect cascading effects on global agricultural production systems. Pandemics cause supply and demand shocks, as well as problems in supply chain management and distribution networks [84]. Stakeholders in processing, retailing, and distribution have been severely impacted by labor issues, transportation issues, and financial insecurity [21]. When it comes to food supply; food quality, safety, logistics and transport are matter of concern while inadequate information, panic purchasing, stock piling, lower purchasing power with declined income are affecting food demand in the food system chain [17, 58, 83, 84].

Addressing poverty, malnutrition, inequality, climate change, socioeconomic disparities, and environmental concerns through sustainable transformation of the food system and supply chains will enhance people's triple bottom lines and reduce health-care costs [73]. The system must be adaptable, equitable, integrated, resilient, healthy, renewable, diverse, and sustainable in order to reap the greatest benefits [122].

**Fig. 6:**
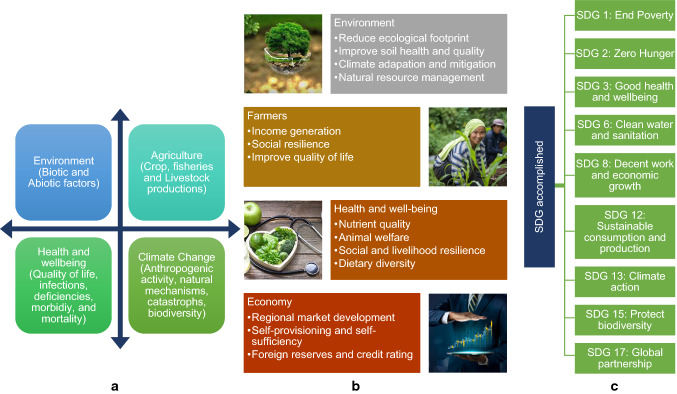
Complex relationships in building transdiciplinary holistic food system resilience. **a** Interacting components, **b** Benefits enjoyed and **c** SDG accomplished. Source [123]

In sustainable food transformation, nexus thinking considers social, economic, and environmental forces to offer a new framework for assessing the complex link between food, nutrient, health, agriculture, and economic growth. Land, water, and energy are all interconnected variables that influence food production. Because of the increased need for production, they are fiercely competing with one another [71]. Complex relationships in building transdisciplinary holistic food system resilience are demonstrated in Fig. 6.

A holistic approach is to be adopted with long term vision to sustain the complex food system framework. Supportive policy frameworks, investment in research, innovation, and development activities, capacity building of farmers and institutions, regular monitoring and updates, customary and consistent assessments, dissemination of new knowledge, practices, and experiences, appropriate technological advancement, and strategic action plans are essential tools in facilitating a positive transformation with social inclusion [73, 92].

African food systems are being transformed using two strategies: climate resilient smart agriculture, multi-stakeholder partnerships, and innovative sustainable agricultural frameworks that prioritize diversified production systems to support livelihoods [95, 122]. Research with small-holder communities has revealed similar findings that diverse food environments in homes and communities enhance food security, diet, and the resilience of the food system. This is demonstrated by a case study in China. The community benefited from wild foods and traditional agricultural techniques during pandemics, and relationships between people and the food environment and people's dependence on food had changed significantly [76].

There is a need to build back better mechanisms in order to develop pandemic resilient food systems [6]. Covid-19 has brought to light the necessity for socio-technical development and innovation in agroecosystem management in order to achieve agri-food system resilience and global food security [50]. The following sound innovative strategies are critical for establishing solid digital platform for paradigm shift to sustain constant food production [21].

### Industry 4.0 technologies

Adoption of industry 4.0 technologies significantly improves the market, supply, finance, infrastructure, production, logistics, environment, policy, regulations, and operation risk management [18]. Artificial intelligence, the internet of things, big data analysis, remote sensing, machine learning, robotics, block chain technology, and digital twins are just a few examples of autonomous decision-making tools that are included [78]. By improving productivity, transparency, and visibility, they increase the resilience of the agricultural supply chain and prevent interruptions in the food supply chains [109].

### Smart and precision farming technology

It is a prospective approach to combat pandemics [124], that reorients the agricultural system in order to improve food security in the face of extreme contexts [122]. Climate-smart precision farming involves use of complete or semi-automation technologies to replace intensive manual labor, innovative designs of mechanical devices such as [78], fertigation drones, satellite data collection and integration with on-farm data for site-specific climatic agriculture, harvest and planting robots, autonomous vehicles such as man-less tractors [63]. It improves the productivity, adaptability, and resilience of the agroecosystem and associated farming community [125].

### Ecological agricultural intensification

Utilizing ecological processes, it relies on efficient management and improved inputs to raise crop productivity per unit area. The rich biodiversity, habitat for wildlife, pest, weed, and disease control, versatility, sustainability, yield stability, healthy soil, microclimate control, and improved nitrogen and water use efficiency are all benefits of this regenerative agricultural mix [23, 104, 111, 126–128].

### Enhancing farm and crop diversity

Diversification strategies implemented at the field, farm, and landscape levels result in greater production to satisfy global food demand [44, 71, 85, 129]; Crop diversification techniques involve crop rotation, poly culture, intercropping, and integrated farming systems such as intensive silvopastoral systems, mixture of local varieties, agro-forestry systems (e.g., Kihamba agroforestry system in Mount Kilimanjaro’s southern slope in Tanzania in Africa)/crop-livestock-fish-poultry-pond-swan integrations [61, 107, 110, 130]. Higher reliance on small number of major cereals is highly risky. The study of Muthamilarasan and Prasad [85] brought out the necessity of diversification of staple crops (millet as a potential new staple crop to be consumed in hunger stricken regions) to endure food and nutritional security amidst pandemics while generating subsistence income to marginalized farming community and enhance diversity [85].

### Resilience farming model

Conventional farming knowledge and techniques are repositories to enhance resilience of modern farming in extreme contexts [110]. Complex indigenous farming systems maintained in Asia, Africa, and Latin America without utilizing modern technologies provides a source of income to smallholder farmers despite of environmental instabilities and variability. Time emerged for the transformation of subsistence agriculture market oriented commercial farming with the advent of pandemics. Agricultural value chain needs to be protected with public private partnership and international funding through global networks, innovative business startups, and mechanization of agricultural productions [57].

### Addressing food security through policy coherence system

Achieving food security and nutrition in the context of sustainable development and pandemic entails inclusive and integrated policy making across multiple disciplines breaking conventional silos. Coherent approach necessitates the diverse stakeholder involvement at local, national, regional, and international levels from both private and government parties [131]. The adoption of international policy instruments like the Paris Agreement, Millennium Development Goals, Sustainable Development Goals, and New Urban Agenda has a significant impact in dealing with climate-related disaster extremes while reducing food-related greenhouse gas emissions. Climate change is the primary factor influencing food production because agriculture is highly climate sensitive. Moreover, it is advantageous to adopt the disaster risk reduction actions plans of the Hyogo framework (2005–2015) and Sendai framework. The advancement of food security, food availability, food affordability, and distribution equity depend on responsible policy mechanisms. Especially the policies related to agriculture (price controls, input subsidy, farm fiscal policy, control of pesticide, regulation of fertilizer, land use policy, and soil conservation), livestock (animal protection ordinance, national animal breeding policy guidelines, animal disease act, conservation and utilization of indigenous animal genetic resources), and fisheries (domestic legal and policy instrument, fisheries and aquatic resource act, international obligation and EU market regulation such as UN fish stock agreement) need to be updated and modified to withstand all types of uncertainty conditions [132]. In addition to these adhering to the intergovernmental policies, treaties, declarations, norms, and standards of global organizations such as UN, FAO, WHO, IMF, World Bank, United Nations, UNDP, UNISDR, and UNFCCC will add consistency to the approach.

### Re-engineering market access

The movement of goods and services between producers, consumers, and businesses is a crucial concern in a pandemic environment. As travel and export restrictions were put in place locally and across trade boundaries to reduce price instability and increase business transparency, trade digitalization, online shopping, and e-business became more common. This has led to an expansion of the global agriculture market [78]. Unlike previously, online grocery delivery and food delivery services (Skip the Dishes and Uber Eats) boomed popular across the world countries [84]. The research examined at the vegetable supply chain in Shanghai and discovered that veggies purchased online are more expensive than those acquired through traditional methods. As a result, vegetable farmers can be encouraged to utilize e-commerce to escape the effects of pandemics and the external environment [120]. This disruption created new opportunities for farmers involved in direct selling, therefore eliminating retail and wholesale as well as new relationships that united farmers and citizens [79].

### Self-reliance agriculture

Following the pandemic, consumers prioritized local food supply chains, which would change the agri-food industry in the long run [84, 95]. In the wake of Covid, urban agriculture is a strategy to enhance food security, nutritional status, end poverty, and build a resilient agricultural system [61, 133]. Locally produced, organic plant-based foods are very nourishing and support a healthy immune system in people [10]. Around 60% of vegetables and 90% of eggs are produced in Shanghai, the largest city in China [68]. Improving local food production necessitates better access to land, water, and other physical and human resources, as well as social inclusion, traditional knowledge and skill transfer, marketing, cold chain storage facilities, and a change in public preference [47]. In Australia, a case study revealed the need for regional knowledge policy, innovation dynamics, planning, design response, and governance response [134].

With the onset of the pandemic, home gardening in Canada experienced a surge in interest as a viable solution to the country's food shortage, as demonstrated by Mullins et al. (2021) using a case study [135]. Self-provisioning is supported by gardens of different types, agroforestry systems, and in particular fruits, vegetables, grains, cereals, legumes, and dairy products [50, 107]. Hernández-Nez et al. [80] discovered that women’s empowerment improves self-provisioning at the local level. With a rise in the number of women per family, technical level, and educational level, species variety and richness in the gardens expanded. It has a substantial negative association with the family's wealth [80].

### Innovative food system governance

Innovative food governance mechanisms must be deployed to accelerate the speed recuperation from Covid-19. The existing food governance need to be reviewed in respect to effectiveness and efficiency considering of available local and international policies, treaties, standards, guidelines, norms, strategies, programs, and legislations related to food security and nutrition [118]. To ensure food security, eradicate poverty, and put an end to hunger in the dynamic context of climate change, socioeconomic limitations, financial constraints, volatile market conditions, political instability, institutional rigidities, and power imbalances, food governance needs to be context-specific and people-oriented. This requires collaborative action from cross-sectoral stakeholder involvement and effective networking. Collective iterative and adaptive learning process and operative institutional frameworks direct to effective long term sustainable practical solutions for food system transformation while balancing the four pillars of sustainability and addressing tradeoffs in policy discourses, resource management, and decision making [83].

## SDG in dealing with pandemic and agroecosystem resilience

There are 17 SDGs, 169 targets, and 232 indicators that international communities intend to eradicate poverty, hunger, and malnutrition while also guaranteeing a healthy and dignified life [77]. Though there is a decade ahead world is still in off-track in terms of accomplishing its sustainable development goals [73, 136]. Covid-19 pandemic further threatens the global efforts [123], but it is critical to highlight mankind's failures and the importance of emphasizing sustainability in decision making, agricultural policy, and action planning in order to fulfill future demands and prevent future pandemics [17, 53]. Among 17 SDGs, Goal 1: No poverty, Goal 2: Zero hunger, Goal 3: Good health and wellbeing, Goal 4: Quality education, Goal 8: Decent work and economic growth, and Goal 10: Reduced inequalities were highly set back by the pandemic. Table 5 provides an idea on SDG targets and indicators setting in progress during and post-pandemic stages.

**Table 5:** SDG targets setting in progress during and post-pandemic stages. (the diagram should be stretched as the SDG goal positioned above corresponding targets and indicators)
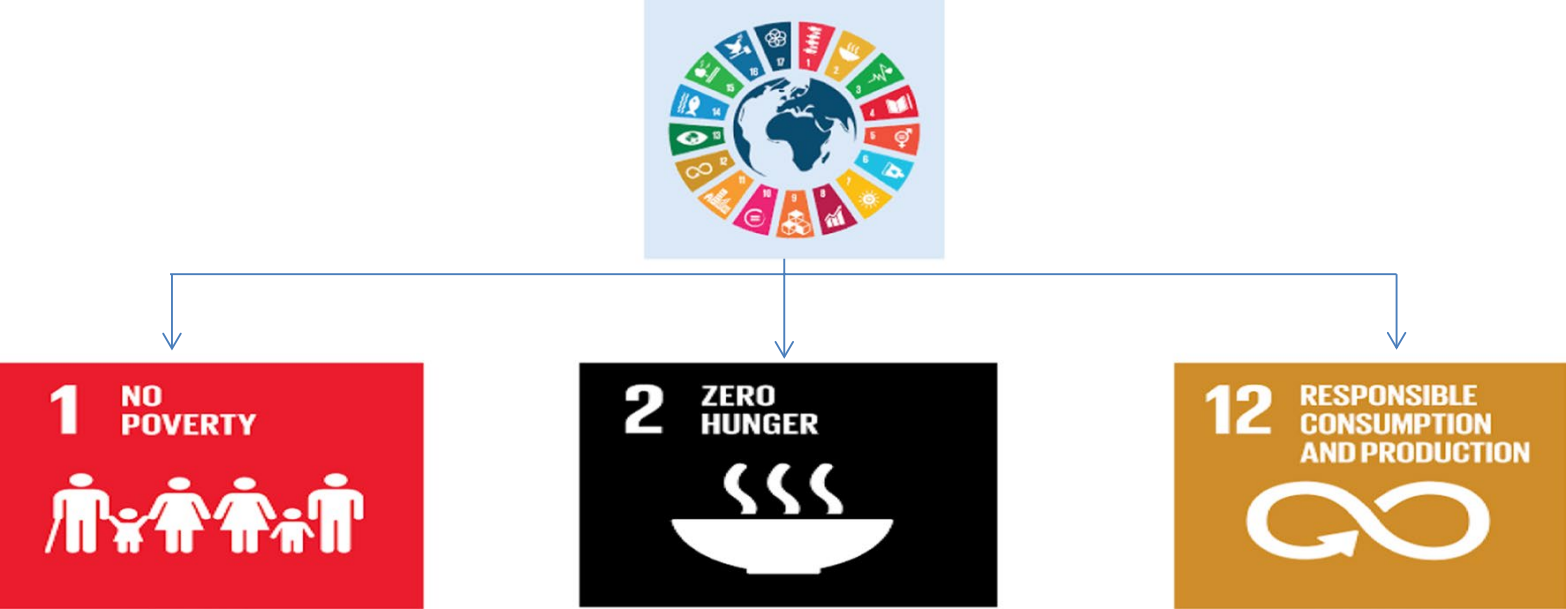 Source: [21, 112, 137–141]

Targets	Indicators	Targets	Indicators	Targets	Indicators
1.1. Eradicate extreme poverty for all people everywhere	1.1.1. Proportion of population below international poverty line	2.1. Universal access to safe and nutritious food	2.1.1. Prevalence of undernourishment	12.1. Implement the 10-year framework of programmes	12.1.1. Number of countries with sustainable consumption and production national action plans, mainstreamed into priority
1.2. Reduce at least by half the proportion living in poverty in all its dimensions	1.2.1. Proportion of population below national poverty line	2.2. End all forms of malnutrition	2.1.2. Prevalence of moderate/severe food insecurity in the population, based on the food insecurity experience scale	12.2. Sustainable management and efficient use of natural resources	12.2.1. Material footprint, (per capita and per GDP)
1.3. Implement nationally appropriate social protection systems and measures accomplishing substantial coverage of the poor and the vulnerable	1.2.2. Proportion of men, women, and children in poverty in all dimensions (national scale)	2.3. Double the productivity and incomes of small-scale food producers	2.2.1. Prevalence of stunting	12.3. Reduce food waste, food losses, including post-harvest losses	12.2.2. Domestic material consumption (per capita and per GDP)
1.4. Ensure equal access to basic amenities, economic, natural, financial resources, and technology without gender and social disparity	1.3.1. Proportion of population covered by social protection floor	2.B. Prevent agricultural trade restrictions, market distortions, and export subsidies	2.2.2. Prevalence of malnutrition	12.4. Environmentally sound management of chemicals and wastes throughout their life cycle, reduce emissions, and adverse impacts on human health and the environment	12.3.1. Global food loss index
1.5. Build socio economic and climate resilience of poor and vulnerable population	1.4.1 People access to basic amenities	2.C. Ensure stable commodity markets and timely access to information	2.2.3. Prevalence of anemia in women	12.5. Reduce waste generation through prevention, reduction, recycling, and reuse	12.4.1. Number of parties to international multilateral environmental agreements on hazardous waste, and other chemicals
1. A. Ensure significant mobilization of resources to end poverty in all forms (for developing and least developed nations)	1.A.1 Poverty eradication assistance programs		2.3.1. Volume of production per labor unit	12.6. Company adapt sustainable practices and to integrate sustainability information into their reporting cycle	12.4.2. Hazardous waste generated per capita, and proportion of hazardous waste treated, by type of treatment
1. B. Develop sound policy frameworks at all levels to end poverty	1.A.2 Government funding on basic amenities		2.3.2. Average income of small-scale food producers	12.7. Promote public procurement practices that are sustainable	12.5.1. National recycling rate, tons of material recycled
				12.8. Information dissemination and awareness for sustainable development and lifestyles in harmony with nature	12.6.1. Number of companies publishing sustainability reports
				12. A. Support developing countries to strengthen their scientific and technological capacity for sustainable consumption and production	12.7.1. Number of countries implementing sustainable public procurement policies and action plans
				12. B. Develop and implement tools to monitor sustainable development impacts for sustainable tourism	12.8.1. Extent to which global citizenship education and sustainable development are mainstreamed in national education
				12. C. Rationalize inefficient fossil-fuel subsidies	12.A.1. Amount of support to developing countries on research and development
					12.B.1. Number of sustainable tourism strategies/policies
					12.C.1. Amount of fossil-fuel subsidies per unit of GDP and as proportion of total national expenditure on fossil fuels

### SDG 1: end poverty

Despite significant advancements in lowering poverty rates and ensuring that everyone has access to food, the Covid-19 pandemic shock caused a reversal in the situation and saw the first increase in poverty rates in decades [142]. 20% more Bangladeshis are now forced into poverty as a result of Covid [119].

The United Nations Covid-19 Response and Recovery Fund raising mechanism intended to tackle health emergency, socioeconomic response, and recovery, and build back better system through integrating shared responsibility and global solidarity in finding urgent solutions to address the needs of confronting population [143].

Foreign direct investment, especially green investments, which foster thriving economic growth and development by luring capital across nations and sectors, is a tool to combat poverty in the face of a pandemic. Through increased employment rates, a reduction in the gender pay gap, and easier technology transfer, economic development is the primary force behind the eradication of poverty. The decline in poverty rates in South America is evidence of this [144].

With the help of world leaders, global poverty projects are attempting to eradicate poverty in order to combat the pandemic [145]. The study illustrated an SDG push scenario that promotes coordinated potential investment in social protection, governance, digitalization, and green economy as a strategy to reduce extreme poverty while outpacing the current progression curves that have lifted 146 million people out of the poverty box [146].

### SDG 2: zero hunger

According to the United Nations Economic and Social Council, 784 million people were undernourished in 2015 [89]. By 2019, it is predicted that 8.9% of the world's population would be (hungry) undernourished [136], stressing the critical need for government assistance for agriculture [49]. According to the World Hunger Index, hunger conditions in many Sub-Saharan African nations are extremely distressing along with the epidemic. As of today, the Covid-19 outbreak is classified transitory food insecurity, which is temporary and short-term but has the potential to cascade into persistent long-term/chronic insecurity [58, 99].

In order to meet global demand, decreased yield and productivity are frequently cited as obstacles to achieving SDG 2. However, it is linked to structural and governance problems like poverty, malnutrition, uneven resource and food distribution, food waste and loss [61, 93, 111]. Additionally, it has a close connection to the development of community resilience, climate change mitigation, and natural resource management [77].

### SDG 12: sustainable consumption and production

The sustainable consumption and production are strongly linked to the development of a resilient food system framework, since responsible transformations in people's food behavior and consumption have a substantial influence on converting food production systems based on potential consumer demand [73]. Between 2010 and 2017, worldwide domestic material consumption has increased by 7% per capita as a result of sustainable consumption and production policies [8].

Due to this pandemic, the responsibility to meet environmental goals has been extended from the hands of government to private, local organizations, community and to individuals. The substantial changes such as online working, e-learning, local productions, responsible consumption, circular economy as a central theory for production, resource efficiency, decoupling production from resource consumption, as well as choices based on lifecycle perspective are all heading to accomplishing SDG 12 targets and are inevitable in the post governance as well [138, 147].

## Policy implications related to COVID-19 in building food system resilience

### Pandemic prevention through health legislation

Policy and governance played a crucial role in controlling pandemic waves. Public health guidelines, national health policies, and emergency laws such as curfews, lockdowns, border closure, banned air travels, limited-service provision, international trade restrictions, closure of public facilities, and prohibited public gathering are implemented globally to prevent the spread of Covid-19 and safeguard public health [15, 57, 58, 63, 115, 120]. Although the laws addressed the public health vulnerability, they gradually lost support, as was demonstrated in Germany by a decline in Covid cases, disruptions to livelihoods, and a severe decline in the economy [4, 148].

Fundamental social and behavioral concerns of Covid-19 include mask policies, social distancing and quarantine policies that are practiced as protective and risk reduction measures against pandemic were successful when implemented as mandatory policies [15, 149].

The “One Health Legislation” policy, an interdisciplinary law that links health issues to produce better health outcomes, aids in the prevention of pandemics by recognizing the interdependence, indivisible nature, and total conservation of the health of the environment, soil, plants, and abiotic and biotic communities [13]. In order to achieve SDG 3 of ensuring health and wellbeing and SDG 15 of ensuring environmental sustainability, this strategy ensures biological and sanitary integrity [2]. Additionally, integrating the “Eco Health” strategies into the responsive mechanism and the “Planetary Health” paradigm, an initiative of the Rockefeller Foundation and the medical journal “The Lancet” to systematize global health [150] are important steps in combating the pandemic [5].

Hsiang et al. [116] analyzed varying policy implementations of six different countries using econometric approach to estimate the impact of anti-contagion policies on the growth rate of infections, the results demonstrated the exponential growth rate (38/ day in the earlier phase) in the absence of policies, thus it is obvious the polices deployed have made a significant contribution in limiting the transmission to a considerable extent while providing measurable health outcomes [116].

### Policy instruments for agri-food system and impacts on trade

Apart from business as usual, a self-sustaining nation requires strong policy implications to preserve food security and sovereignty even in times of epidemics and pandemics. With the introduction of Covid-19, the foundation of policy formulation has shifted to include the resilience and adaptability of agricultural food systems to deal with all types of normal and abnormal future consequences. To deal with unexpected crisis situations, world countries had shifted their focus away from developing and modifying existing food safety policies [78].

Coherent and effective health and nutritional policies also implemented to avoid high calorie and unbalanced diets in EU nations, but the execution needs to be more effective to curtail global pandemic shocks and enhance nutritional quality of diet [5]. The biodiversity for food and nutrition initiative in Sri Lanka, Brazil, Kenya, and Turkey has incorporated agricultural diversity into national policy changes to enhance human health and food security that are highly beneficial to deal with pandemic [8].

Policy development that addresses social, environmental, economic, and gender equality improves agricultural productivity and resilience [63]. Agriculture and economic policies must prioritize the development of farmers’ and production companies’ capacity to manage and withstand external turbulences and volatile market conditions [124]. International and national collaboration to generate resilient policies will favor escalating process of agriculture capacity. Besides, carbon credit, farmer remuneration for carbon sequestration while phase out fossil fuel subsidy, external inputs, industrial nitrogen fixation are potential legislations to overwhelm pandemic [113].

Timely implementation of business and trade policies including export ban, export quota, import tariffs, quality control, and quantitative control, and tax policies supported many nations to tackle the economic down fall, inflation, GDP decline and managed to maintain the foreign reserves ensuring favorable operating environment for credit rating. Immediately after Covid-19 outbreak most of the governments declared the state of emergency and involved in emergency financing. According to IMF, liquidity, and solvency policies support to uplift households, business, and financial sectors. Solvency measures encompass cash transfers, unemployment insurance, equity injections, subsidies, and government guarantee while, liquidity measures include loan or deferrals such as suspension of mortgage loans, extension of maturity period, credit guarantee, and credit provision. In addition to these, rationing, and price controls, acts against hoarding are essential in managing extreme food shortages [151].

Central Bank of Sri Lanka had eased its monetary policy with historically low interest levels, concessional financing, subsidy schemes, refinancing, and credit guarantee schemes while, emphasizing the need of robust monitoring and market regulation for liquidity measures to ensure food security and better functioning of food system governance due to the market power of intermediaries [152].

Most developed countries have implemented monetary policies to mitigate pandemic shocks, but the stories in developing countries are quite different. To work out the economic response, they need a national response plan and centralized crisis management. Unprecedented disasters, such as Covid-19, necessitate prudent unconventional responses from the international community in order to limit global damage and assist developing nations in recovering. International actions from G20 and UN can support the nations through unconventional monetary policies in conjunction with fiscal stimulus in developing nations, UN and Multi-Lateral Development banks (MDB) can advise in policy responses, co-funding with local private banks or investors, establish mechanism for debt resolution, provide special drawing rights, and enhance the lending capacities to strengthen the social safety nets, food supply chain networks, increase budget allocation for health care, and revival of small and medium sized enterprises [153].

## Conclusion

**Fig. 7:**
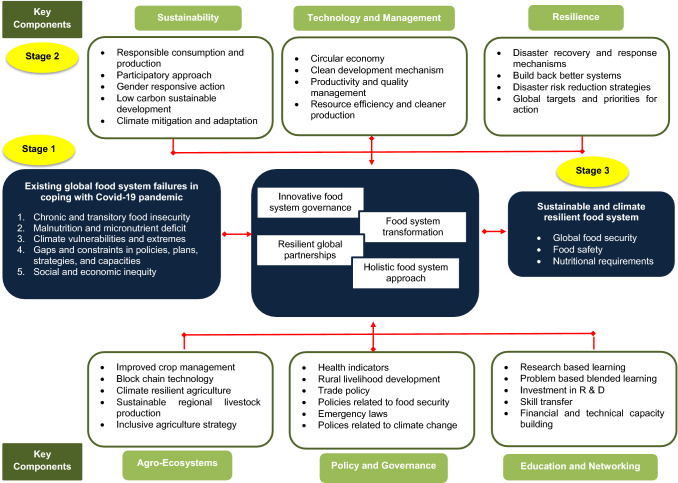
Conceptual Framework on ensuring global food security and building pandemic resilient food system: towards accomplishing SDG targets

A conceptual framework (Fig. 7) for future perspectives on ensuring global food security and building pandemic resilient food system towards accomplishing SDG targets has been developed. In order to address future global emergencies, the framework emphasizes the necessity of an inclusive and integrated approach spanning multiple disciplines with cross-sector stakeholder involvement at various hierarchical levels. A number of social, economic, environmental, cultural, technical, and management factors were taken into account when developing the framework, and the literacy in the food system, agriculture, climate change, resilience, and policy development was also included. There are three stages in the development of framework; first is the identification of existing global food system failures that create substantial barriers in coping with Covid-19 pandemic and achieving global food security through extensive literature survey. Malnutrition, a deficiency in micronutrients, climate vulnerabilities and extremes, gaps and limitations in policies, plans, strategies, and capacities, as well as social and economic inequality, are the root causes that have been identified. The second stage's key elements and corresponding strategies or action plans that influenced the transformation of novel foods were then qualitatively examined. They include sustainability, technology and management, resilience, agroecosystems, policy and governance, and education and networking. These narratives prioritized for a holistic food system transformation are not only to deal with the bounce back from pandemic but also to ensure sustainable future with zero hunger and no poverty. In the final stage sustainable and climate resilient food system is built ensuring global food security, food safety, and nutritional requirements for a healthy living through bridging the gap in accomplishing SDG targets.

To ensure global food security and the sustainability of the food system, special attention is also required in the following areas.

- Increase the effectiveness of specific social protection programs to enhance access to healthy and nutritious foods [22, 51].
- Improve safeguards for poor and marginalized food system workers, farmers, and women who have been disproportionately impacted by the crisis [24, 110, 154].
- Improve safeguards for nations that rely on food imports [124].
- Aid in the development of more diversified and robust distribution networks, including shorter supply chains, circular economies, and territorial markets [95].
- Encourage more robust food production systems based on agroecology and other sustainable food production methods [3, 70].

## Data Availability

Raw data is available upon request from the corresponding author.

## Code availability

Source code is available upon request from the corresponding author.
